# Abstracts and Gleanings

**Published:** 1879-01-20

**Authors:** 


### ABSTRACTS AND GLEANINGS.

### REVIEW OF THE PAST YEAR.

In medicine and therapeutics much progress has been made. The question of blood-letting is still being revived from time to time. Wharton Jones, in the *London Lancet*, Nov. 2d, gives his views regarding the subject. H thinks by the prevailing abstinence from venesection inflammation of important organs is often allowed to run a prolonged and disastrous course, which might be prevented by the timely abstraction of blood in such quantity as could not be injurious to the patient.

**Transfusion.**—The subject of transfusion has also been debated, and experiments have been performed by Dr. Brown-Sequard, of Paris, with different fluids. He tried normal blood, blood deprived of fibrin, and milk. In each case he found the result to be the same, but the quantity of milk used was greater than the other fluids. He considers it preferable to inject into the arteries rather than the veins, and to be done slowly. Dr. Thomas, of New York, has also tried the transfusion of milk, and is convinced that it acts as well as blood.

**Night Sweats.**—Atropine as a remedy for night sweats in phthisis has been brought into favorable notice. It has been tried in the Toronto Hospital with uniformly good results. The dose is about one-fortieth of a grain of the sulphate of atropine at bed time. Chloral hydrate still continues to be used with great success in the treatment of delirium tremens. Dr. Farrar in the *Brit. Med. Journal* for January, 1878, speaks in the strongest terms of its beneficial effect after the failure of the opium treatment. The subcutaneous injection of ergotine is now almost universally considered a sovereign remedy for hemoptisis, hematemesis, uterine hemorrhage, etc. Its *modus operandi* depends upon its action on the vaso-constrictors. Where there is much pain or irritable cough, it is combined with morphine.

**Dialyzed Iron.**—Dialyzed iron, which has been so favorably received by the profession, has lately been used by way of hypodermic injection. Prof. Da Costa, of Philadelphia, has tried it with marked success in several cases of anæmia and chlorosis. The iron was used in both the diluted and undiluted state, without any unpleasant effects. It was injected in from fifteen to thirty minim doses daily. The muriate of calcium has been highly extolled by Dr. Bell (*London Lancet*), in the treatment of tuberculosis. He gives it the preference over all other remedies in the treatment of this affection. He has used it also successfully in scrofulous disease of the bones, tabes mesenterica, etc. The dose is 20 grains, more or less, after meals. Iodide of ethyl has been employed by Prof. See for the relief of the paroxysms of asthma. It is administered by inhalation, a few drops being placed on a handkerchief and applied to the nose.

**Nitrite of Amyl.**—Nitrite af amyl, so much vaunted of late to a remedy for sea-sickness, has been used to cut short the cold and has stages of ague, and thus facilitate the treatment. Some experiments were performed in the Greenwich Hospital by Dr. Ralfe with such apparently satisfactory results as leads us to hope that with a more extended trial its efficacy may be established. The use of carbolic acid in small-pox has been frequently alluded to in the different medical journals during the year. It has been given internally in small doses and also used as a lotion, in the proportion of 1 in 20, applied to the face. It seems not only to act as a disinfectant, but also to prevent the pustules becoming confluent and to moderate the occurrence of pitting.

**Nux Vomica.**—Nux vomica has been lately used with success in Italy in the treatment of diabetes. Two cases are reported as having been cured by it, in the *Gazetta Medica di Roma*, under the care of Dr. Zarzana. The process of tapping for dropsy of the limbs, first introduced by Dr. Southey, has been put into practice with favorable results. It consists in the introduction of minute canulæ made of gold, which gradually drain away the serum lying among the tissues. To the extremity of the canulæ are attached slips of rubber tubing about two feet in length to convey the fluid into a vessel for its reception. Capillary tubes have also been used for paracentesis abdominis and thoracis by Dr. Goodhart, of Guy's Hospital.

**Metalloscopy and Metallotherapy.**—The subject of metalloscopy and metallotherapy has engaged the attention of scientific physicians, in the treatment of hystero-epilepsy, in which anæsthesia or hemianæsthesia are frequent symptoms. It consists in the application of certain metals varying according to the idiosyncrasy of the patient, the process of ascertaining which is called metalloscopy. Bits of metal are applied to some part of the surface on the anæsthetic side. A piece of metal is also introduced into the mouth or applied over the mastoid process. If the proper metal has been hit upon, the sensibility is restored wholly or in part; if not, another and another kind of metal is tried until the proper one is ascertained, which is then kept applied, or some salt of the metal administered. The metal is supposed to act by creating a current of electricity which affects the vaso-motor nerves, so that an increased blood-supply is sent to the parts.

**Artificial Respiration.**—The direct method of artificial respiration introduced and practiced by Dr. Benjamin Howard has been on its trial during the past year. The claims of superiority over Marshall Hall's or Sylvester's method, are its simplicity and the readiness with which it can be employed by water police and others who may have once seen it put in practice.

**Surgery.**—In the domain of surgery much has been accomplished, and several new and important procedures have been introduced into practice. A new departure in the operation of lithotrity has been brought forward by Dr. Bigelow, of Boston. Instead of short and repeated operations, he recommends longer time and an attempt to break up the stone, and wash it all away by a contrivance for the purpose, if possible at a single operation. Thus far, the success of the prolonged operation has been very good. An improvement has also been introduced in the operation for removal of the tongue in cancer, by Dr. Shrady, of New York. It consists, first, in ligaturing the lingual arteries at the posterior border of the hyo-glossus muscle, after which the removal of the tongue, it is claimed, is almost bloodless.

**Catgut Ligature.**—The results obtained with the catgut ligature have been conflicting. Some surgeons have found it uniformly effectual and safe, others have met with only disappointment and disaster. It is liable to soften and the knot to unloose, and thus permit of secondary hemorrahage. The use of the elastic ligature for the division of the prepuce in the treatment of phymosis has been tested in several cases, and with very good results. It is applicable in a number of cases where circumcision or the use of the knife is inadmissible.

**Removal of Rectum for the Relief of Cancer.**—The operation for the removal of the lower end of the rectum for the relief of cancer, has been several times performed during the year, and quite recently by Dr. Fenwick, of Montreal. The success which has attended the operation so far, is such as to encourage a repetition in certain cases where there is reason to believe that the disease can be surrounded and completely removed by the knife. The control of the bowel afterwards is wonderfully good considering the nature of the operation, and in those cases in which the sphincter action is impaired or lost, the patient still retains the sensation of the presence of fæces, and can make preparation for cleanliness.

**Removal of the Spleen.**—Another case of removal of the spleen has been put upon record by Dr. Browne, of the Bromwich Hospital. The tumor, which proved to be a simple hypertrophy, weighed 18½ pounds. There was no hemorrhage. Four large arteries required the ligature. The patient, previously very much reduced—in an almost hopeless condition—died five hours after the operation. The treatment of varicose and other chronic ulcers of the leg, by the elastic bandage, has been successful in the hands of Dr. Martin, of Boston, and others who have tried it The bandage is of pure rubber ten feet long and three inches wide, and is applied as an ordinary bandage to the leg. It is removed at night, and the ulcer dressed with suitable dressing.

**Aneurism of the Aorta.**—The treatment of aneurism of the aorta by the subcutaneous injection of ergotine conjointly with galvano-puncture has been tried, with very favorable results, by Dr. Carter, of Queen's Hospital, Birmingham. Two needles connected with a Stohrer battery were used, first commencing with two, and afterwards increasing to twelve cells immersed half way in the acid. The operation was twice repeated at the end of a week or ten days, in each interval. The needles were allowed to remain in the sac from 35 to 50 minutes at each sitting. The result exceeded the most sanguine expectations, the patient being very much relieved, though not completely cured. Excision of the bones of the foot for the cure of talipes in the adult, has been successfully resorted to by Dr. Bryant, of Guy's Hospital, and others in Europe and America during the past year. Two cases o gastrotomy have been reported. One successful case by Prof. Trendelenburg, of Rostock, in a boy eight years of age, and an unsuccessful one by Dr. Bradley, of Manchester, in a boy 14 years of age. Several successful cases of paracentesis of the pericardium have been reported in Europe and America, so that the operation has come to be regarded as a perfectly legitimate and favorable one in certain cases. A small aspirator needle is that generally used, and no difficulty has been experienced in the operation. The needle is introduced in the fifth intercostal space, nearly in the position of the normal apex-beat. Aspiration of the knee joint has been several times performed—in one case in the Toronto general Hospital under the writer's care—with the most beneficial results. If performed so as entirely to exclude the air, it is perfectly safe. Prof. Langenbeck successfully extirpated the left kidney of a woman aged thirty-two years. This operation has also been successfully performed by Dr. Martin, of Berlin.

**Laparotomy.**—The operation of laparotomy has been resorted to by many surgeons on the Continent, both for the relief of disease and for the purpose of clearing up the diagnosis in obscure cases. The abdominal cavity is now opened with as little hesitation as that with which the ordinary surgeon would open an abscess. Gussenbauer, of Luttich, performed the operation of resection for intestinal obstruction, removing four inches of the lower part of the descending colon and a tumor which was the cause of the obstruction. The patient died from septic poisoning, caused by the escape of the contents of the bowel into the abdominal cavity.

**Thymol.**—The new antiseptic thymol has received marked attention among British and Continental surgeons during the past year, and bids fair to supplant carbolic acid as the most available surgical antiseptic we possess. It is the essential principle of the oil of thyme, and is a more powerful antiseptic than carbolic acid. It is not so irritating, and in antiseptic surgery its advantages over carbolic acid are most marked. Spencer Wells has used it in a series of ovariotomy cases with the most satisfactory results. The strength used is one gramme (15 grs.) of thymol, to one thousand grammes of warm water. It does not in the slightest degree interfere with the healing process.

**Obstetrics and Obstetrical Surgery.**—In obstetrics and obstetrical surgery we note a few novel features. In the treatment of vomiting of pregnancy, much benefit has been derived in many instances from the topical use of caustic applications to the cervix uteri. In some cases, a single application of the caustic was sufficient to allay the most distressing vomiting. Laparo-elytrotomy has been resorted to in several cases in the United States, by Prof. Thomas and Dr. J. C. Skene, and once by Dr. Hime, of Sheffield, England, as a substitute for Cæsarian section. The operation is performed by making an incision in the abdomen from the anterior superior spine of the ilium to the spine of the pubis. The peritoneum is drawn upwards, a probe is introduced into the vagina, which is pushed upwards into the bottom of the wound and divided. The os is reached, the hand introduced, and delivery effected by turning. The advantages are the peritoneum and uterus are not wounded, there is very little loss of blood, and the shock is less than in Cæsarian section. The use of hot water in surgical cases and in uterine hemorrhages, has been still further put upon its trial. In some severe cases of uterine hemorrhage, it was found to produce immediate and energetic contraction of the uterus. The temperature of the water should be about 120° F.

The following case is novel, and if the cure should be permanent, will have an important bearing on operative gynæcology in relation to disease of the uterus. We do not know of a similar case on record. It is a case of enucleation of the uterus per vaginam for epithelial cancer, reported in the *Pacific Medical Journal* for Dec., 1878. The operation, which was successful in its results so far, was performed by Prof. Lane, of the Medical College of the Pacific. The uterus was well drawn down, and the process of enucleation commenced by severing with a blunt dissector and pair of scissors the tissues immediately surrounding it, avoiding the rectum and bladder, until the fundus was reached, when the Fallopian tubes were divided and the uterus removed. Several ligatures were applied to the bleeding vessels. The patient made a rapid and satisfactory recovery.

In chemistry we have to record the brilliant discovery of Mr. Piquet of the liquefaction of oxygen. Under a pressure of 300 atmospheres and the influence of intense cold, oxygen becomes a liquid. Lately, the important discovery has been made by Norman Lockyer and communicated to the Academy of Sciences in Paris, that many of the so-called elementary bodies are in reality compounds, and that some of the metals are interchangeable at very high temperatures.—*Canada Lancet.*

---

**Modus Operandi of Skin Grafting.**—Pinch up a small amount of the cuticle from the inside of the arm with a small pair of forceps, and divide it with a small pair of concave scissors, being careful not to draw blood, and get the slip free from adipose tissue; insert this piece of skin into the granulations about one inch from the margin of the sore, and repeat it in a similar manner until you have slips within an inch of one another all over the abrasion. The size of the piece of cuticle is not so material; the object to be attained is to have it to grow; and it cannot take root unless it be buried into the ulcer. A piece of skin the size of a pin's head is large enough. When the grafts are all inserted, dress the sore with an artificial scab, made of adeps porci, nine ounces, and thickened into a paste with English prepared chalk, twelve ounces, and spread over the sore and margins, retaining it there with a roller or adhesive plaster. Let this dressing remain on for three days, then redress by removing the artifical scab, and wipe the sore with a soft cloth. Right here, gentlemen, permit me to digress by saying, never use any water in dressing old sores, for it seems to make them take on those horrible nocturnal neuralgias the night following. Then remove your paste carefully and wipe dry; wherever a slip has taken you will notice a small depression at that point, and if you think advisable you can insert other grafts, and dress as before, and so on, until cicatrization is perfected.

Until the sore is studded full of grafts, and the ulcer, to all appearance, about well, I would suggest, if the sore is on the lower limb, quietude in the recumbent posture, and the leg elevated, as any pressure upon the ulcer at this time would cause most serious interference with the new granulations, as well as destroy the newly-formed cuticle within the sore, which resembles so many small islands in a body of water; these little islands of skin will meet the skin from either side, and thereby bridge the abrasion over. By carefully watching at this stage the new skin will become permanent, so that your patient may be allowed to go about. If the sore is on an upper extremity, the patient can have more liberty to go around. As regards the dietetic plan in these cases, as a general thing, the regimen should be articles of diet containing fat nitrogenized, and phosphatic combinations, together with milk and eggs. Stimulants should be avoided.

*Report of two Cases.*—Mrs. B., aged sixty years, has had an indolent and irritable ulcer covering the dorsum of both feet and extending up the ankles, involving both malleolus processes, with indurated edges around both sores. This woman has suffered considerably, as her present physical condition shows, and she has tried a legion of remedies, as well as a goodly number of physicians, during her long period of illness, but all to no purpose, so far as giving her any permanent relief. The feet would, at times, under a soothing treatment, heal over about one half of the abrasion.

In December, 1875, her husband sent for me, and after taking in her situation and the character of the ulcer, I made the following prescription, viz.:

| | | | |
|---|---|---|---|
| ℞ | Phosphoric acid, diluted, | f ʒss | |
| | Glycerine, pure, | f. ʒj | |
| | Fowler's solution arsenic, | gtts.iij. | M. |

SIG.—To be taken three times a day after eating.

Locally I used elm poultices every six hours, for two days. Then I began my favorite treatment, in order to give tone as well as a healthy appearance to these sores, consisting of:

| | | | |
|---|---|---|---|
| ℞ | Cinchona pulv., | ℥j | |
| | English prepared chalk, | ℥j | |
| | Hyd. sub. mur., | ʒj. | M. |

SIG.—Dust the ulcer and margins over twice a day with this powder.

In a forthnight I had the pleasure of seeing the sore and the surrounding surface on the same plane, with all pain abated, and my patient comfortable.

I then began skin grafting, by taking the slips of cuticle from her huband's arm, and inserted about twelve points in each sore at my first operation. I then applied an artificial scab, as before described and encased the whole with a roller of bandages. I repeated the dressing in a similar manner twice a week, and inserted skin grafts, as I thought advisable, on each visit; in all I must have inserted not less than 100. At the expiration of three weeks, I discontinued inserting grafts, because there had taken some ten or twelve points in various places in each ulcer; I simply used the artificial scab, with bandaging, and added one drachm of iodioform to each dressing. After six months' perseverance I had the pleasure of seeing both feet heal up entirely, and they remained so for about one year, when the right one broke out again, in consequence of a stick of wood falling upon it endwise, inflicting an incised wound, about two inches by one inch. This has not healed as yet, but she suffers very little inconvenience with her feet at present.

*Case* 2.—Mr. W., aged 25 years, had a large abrasion of the cuticle, about eight by five inches, caused by a contusion of the calf and the skin of the right leg. This sore had been discharging for something like a year or two, with no apparent hope of cicatrization. When I was called to take charge of the case I found the patient's general health good, but the sore was an extensive one. My first dressings, for three or four days, were chalk, Peruvian bark and calomel, as before described. I then wiped the sore dry and inserted five skin grafts, and dressed as in the first case. In three days I again inserted three more grafts. At the expiration of three weeks I had the pleasure of seeing cicatrization perfected. This man went to work in less than six weeks, driving a team for one of our grocery men. His leg is still well.—DR. GAINSON, in *Med and Surg. Reporter.*

**Morphia-mania**, or the disorder induced by the habitual use of morphia, has, perhaps, very little claim to notice in a summary of the advances in the practice of medicine; yet, in consequence of the rapid increase in the consumption of opium and its various preparations, both as a means of cure and palliation in disease, and as a vice becoming more and more prevalent in every grade of society, we may expect, ere long, to see the disorder asssigned an appropriate place in every work on Practice.

As regards the evils resulting from the excessive use of opium, while they are not, perhaps, so apparent, and may be less in extent than those attending the use of alcoholic liquors, in effect they are doubtless but little less. The real and fancied wants of suffering humanity, the encouragement given too often, we fear, by our own unguarded prescriptions, together with the facilities afforded by every country store for obtaining the drug, are so many incentives operating in favor of its use; and unless legislative action will assume, in some way or other, to restrict its indiscriminate sale, it may be found not far hence to be a most formidable national evil.

Prevention here will not only be better, but easier than cure; and the profession may, in the absence of other things, effect much good by discouraging, as far as personal effort and influence will go, its too frequent use in the higher forms of disease, and always when taken as an habitual stimulant.

When the habit is once formed, rescue is difficult. Proressor Leidesdorf, in a paper read before the International Royal Society of Vienna, reports the results of his observations in five cases of morphia-mania which had occurred in his own practice in the course of the last year.

The quantity of morphia used hypodermically was from 6 to 37 grs. daily. The sudden withdrawal of the morphia produced diarrhœa, mental irritation, persistent loss of sleep with hallucinations, melancholia, increased sensibility and collapse.

The length of time the drug had been taken in each case is not stated. The treatment consisted in a gradual withdrawal of the opiate with hydrate of chloral, wine, warm baths, persuasion, restraint, watching, etc., and resulted in curing the habit in from eight days to eight weeks. One case relapsed and committed suicide.

Dr. Leidesdorf says: "The patient must be entirely under the control of the physician, as deception is otherwise certain; all the patients had their syringes and a supply of morphia for several weeks. A careful watch must be kept up during the treatment (eight or ten days), to prevent attempts at suicide."

Dr. Winternitz says that "in cases of morphia-mania the lowering of the temperature by baths to 68° to 77° was borne ill or not at all; while, on the other hand, the application of hot cloths along the cervical spine had some effect in reducing the desire for morphia. (Extract from *London Medical Record* in *Abstract of Medical Sciences*, *January* 1877).

Absolute control of the patient is a *sine qua non* in the treatment, and this can rarely be obtained outside of a well arranged hospital, to which such patients, as a rule, ought always to be sent. Not the patient alone, but anxious and sympathizing friends, are as little to be trusted sometimes as the patient himself.—*Dr. Apperson Transactions of Virginia Medical Society.*

[A confirmed opium eater is seldom cured. It is believed that the opium antidotes, so-called, all contain opium. The best thing that can be done is to taper off the habit gradually, sustaining and toning up the system with quinia and nux-vomica as the opiate is withdrawn. We have done this, and discharged our patients after months of careful attention as cured, but *in every case* that has come under our knowledge, the patient has gone back to the habit again.—ED. R.]

**Ice Cream Poisoning.**—In the June (1874) number of the *Virginia Medical Monthly*, there is an interesting account of a paper presented by Dr. L. Rosenthal before the Berlin (Germany) Medical Society, and the subsequent discussion in regard to a considerable number of cases which occurred in that city, which were attributed to poisoning by vanilla, which had been used as the seasoning, although it was admitted that the same seasoning was very largely used in the preparation of tea and chocolate as well as sauces and syrups without any such poisonous effects. There the cases which were quite numerous were all traced to one particular *cafe* called the Vienna, and were during the latter days of August. The article used was what was called vanilla ice, but is described as follows: "In the preparation of cream ice, eggs and cream are used and the flavor is given by adding vanilla, chocolate, etc. Besides these, the various kinds of cream ice are boiled before being introduced into the freezing vessel." The discussion showed that the same night a very large number of persons had been affected by the article obtained from this same *cafe*, and the confectioner exhibited the pods of vanilla which were the same kind as those he had been using for some months without any poisonous effect. The explanation given was that it was produced by the vanilla beans, and especially those which were covered by crystals; but there is no statement made of the composition of these crystals. Now, vanilla is the most popular seasoning and is daily used; and if it were liable to produce such effects, we should see such cases every day. But this very series of cases are commented on by Taylor in his standard work, 3d edition, on *Poisons*, in the following words: "But vanilla ice had been frequently eaten before the outbreak of cholera in 1873 without such severe symptoms, and it is highly probable that the medical man was misled by the number of persons who were attacked simultaneously. *There is nothing in vanilla to cause potsoning.* It contains a crystallizable principle, vanillin or vanillic acid, which is not poisonous (*Hauseman's Pfhauzenstoffe*, 1871, p. 1038). In order to account for the irritant effects, Dr. Rosenthal believes that there was a production of cardol—an irritant oily principle (found in anacardium occidentale), somewhat resembling cantharides. The facts are more consistent with the effects produced by malignant cholera."

From a careful investigation of all the facts, I have come to the conclusion, that the true cause is some decomposition in the albuminoid articles used, viz: the milk and eggs which may be aided perhaps by the sugar. The symptoms described as having been produced by cheese are so exactly like those in these cases, that we may fairly assign them to a similar cause; and I learn, by a private letter from Dr. Kedzie, the able President of the Michigan State Board of Health, that he has analyzed several specimens of cheese which had caused similar symptoms without detecting any mineral poisons whatever. Nearly all these cases occurred in hot weather, and in most the article had been kept some time. Besides, when we reflect how liable milk is to become impregnated with medicinal and other substances by the food and ingesta of the animal furnishing it, I think we may more justly attribute the symptoms to the milk than to the flavoring.—*Transactions Medical Society of Virginia*, 1877.

[If any of our readers know of cases of ice cream or custard poisoning, in which the cause was discovered, they will please report the same to this Journal.—ED. RECORD.]

**The Use of Jaborandi at Bellevue Hospital.**—Within the past year or two jaborandi has become a very popular and useful drug at Bellevue. In uræmia and in acute and chronic parenchymatous nephritis, it has accomplished especially good results.

In uræmia it is a very effective substitute for the old hot-air bath, acting more quickly and surely. As it has been shown to increse markedly the excretion of urea, it is probably more efficient also than the baths in relieving uræmic phenomena. A patient was brought into the hospital some weeks ago, suffering from convulsions and delirium. She had no œdema, but her urine was nearly solid with albumen, and contained small casts and blood. She was given a drachm of the fluid extract of jaborandi, hypodermically, and m. x. of Magendie's solution. In fifteen minutes she was sweating profusely, and the convulsions had ceased. She was restless and wandering in mind for the next twenty-four hours, but had no other bad symptoms. A drachm of jaborandi was given every other day subsequently, and in a week the albumen had nearly disappeared from her urine, and she felt quite well.

Cases of chronic nephritis have been treated with the drug very satisfactorily. Some who did not improve or get rid of the œdema under digitalis and potassium have shown immediate improvement under jaborandi. It is given in drachm doses every other morning, the patient being kept in bed until dinner-time, when the sweating is over. It is better not to give it at night, as the bed-clothes become saturated with perspiration and sleep is disturbed and uncomfortable.

Jaborandi weakens the heart. It is dangerous when the pulse is poor and the system debilitated. If given to a patient in this condition with uræmia, he falls into a cold perspiration, and œdema of the lungs, coma and death follow.

Yet it has been used several times in the treatment of pulmonary œdema in doses of m. x. to m. xv. every one or two hours. The autopsies have shown the usual changes.

It has been used also in pleuritic effusions, but does not seem to "sweat out" the intrathoracic liquid very much. Besides, it produces a nausea and salivation not at all pleasant.

The drug loses its effect in some cases, and the dose has to be increased. The usual variety in its action has been noted. Sometimes it causes salivation only; most frequent salivation and diaphoresis. If the dose is carefully regulated, nausea and vomiting need not be a frequent complication. The urine is, in cases of chronic Bright's disease, somewhat diminished in amount, unless renal congestion or an acute nephritis is complicating the case. Jaborandi has proved, so far, of most certain service in the chronic stages of Bright's disease and in uræmia brought on during its initial attacks. When an acute attack is lighted up on a chronically inflamed organ, and when the system has already become weakened and anæmic, the drug may be useful, but it will also be dangerous.

**Incubative Period of Scarlet Fever, and of Some Other Diseases.**—Dr. Murchison read an abstract of a paper upon this subject at a recent meeting of the Clinical Society of London. The article was based upon observations made in seventy-five cases, and extending over a period of twenty years. Observations upon the incubative periods of small-pox, varicella, measles (about ten days), whooping-cough, typhus fever (supplemental to observations published some years ago in the *St. Thomas' Hospital Reports*), enteric and relapsing fever were given, but with the result that in none of these diseases was the period of incubation fixed. The eruptive fevers in this respect fell, however, into two classes: one class comprised variola, varicella, measles, typhus, enteric fever, relapsing fever, mumps; these had a long period of incubation, whilst erysipelas, diphtheria, dengue, and scarlet fever had a short incubative period; that of scarlet fever is variously given, at from two days to one month, but Dr. Murchison published in the *Lancet*, in 1864, an analysis of twenty-three cases, in none of which did it exceed one week. The present paper comprised twenty-five cases, in two of which (cases of two children on shipboard), the incubative period was very short, in one not exceeding eighteen hours, in the other being less than twenty-four hours. In the well-known case at the West End, some few years ago, where guests at a dinner party were subsequently attacked by scarlet fever, there was no incubative period longer than five days. In fact, in none of Dr. Murchison's seventy-five cases was the period longer than six days; in forty-four it did not exceed four days, in sixteen it did not exceed two days, and in fifteen it was not over twenty-four hours. Dr. Richardson's personal experience, Trousseau's case, in which the incubative period was seven or eight hours, and other well-recorded cases of short incubative periods, were given. The conclusion drawn from these cases is that if a person, after exposure to scarlatinal infection, be subjected to a week's quarantine, and in that period shows no symptoms of having been infected, he is safe. Dr. Murchison has adopted this rule, and has never known it to fail. With regard to the point whether it is possible for the patient to communicate an infectious disease during the incubative period, Dr. Murchison gave some instances of small-pox, which render it probable that such might be the case.—*The British Medical Journal, June* 29, 1878.

**Enemata of Chloral in Sick Headache.**—Dr. J. Seure (*Bull Gen. de Therap.*, 1878, p. 365) recommends this treatment very highly. He says that a patient of his, a lady, who is subject to severe attacks of migraine after shopping, etc., is accustomed, on her return home, to take an enema consisting of a glass of warm water, with a teaspoonful of the following mixture: ℞ chloral, gr. xlv; aq. distillat., f℥x.—M. She then reclines upon a sofa, with closed eyes. Within a few *seconds* she begins to taste the chloral in her mouth, and at the same time she experiences a sensation of numbness. Little by little the headache disappears, nausea is allayed, and half an hour later nothing remains but a slight discomfort in the head, with a little torpor.

Within an hour and a half this lady finds herself able to sit down to dinner, and by the time the meal is over she has forgotten all about her headache and is able to entertain visitors during the evening. In this case twenty grains of the chloral are enough, but in the case of men thirty to forty grains are required. Dr. Seure has noticed that the relief gained is more prompt if a table-spoonful of brandy or whisky is added to the enema. The enema has one disadvantage; that is, the slight burning pain which it causes in the rectum. This may be avcided by the use of a glass of warm milk instead of water, or better by beating up the yolk of an egg in the water. In the case of individuals who retain enemata only with difficulty, a smaller amount may be injected, and a drop or two of laudanum may be added. Dr. Seure regards this treatment as almost infallible for the arrest of an attack of sick headache, and as decidedly preferable to the administration of remedies by the mouth. It has the advantage of not disturbing the stomach. Chloral also acts very promptly, its absorpion by the rectum being almost instantaneous, as is proved by the effects on the general system, and also by the exhalation of chloroform by the lungs within a few seconds after the enema has been taken.—*Medical Times.*

**Inflammation of the Bladder.**—The best remedies to administer internally when vesical irritation and inflammation exist, are gelseminum, belladonna, sulphate of magnesia and pinus canadensis. If the pain be great, choose gelseminum; if the irritation will not admit the presence of a teaspoonful of urine in the bladder, give small doses of sulphate of magnesia; if too much urine be secreted (diabetes), administer pinus canadensis; if the kidneys secrete irregularly. belladonna is indicated. It is not to be supposed that no other agents are "specific" in cystitis, for every experienced practitioner knows of others. However enough have been mentioned to begin with.

Such agents as are known to be diuretic in their action should not be administered in cystitis; better give those agents that tend to restrain urinary secretion. Spices are especially to be avoided. A man or woman having cystitis is made worse by taking stimulants and aromatics. Gin is occasionally prescribed in urinary troubles, but oftener with bad results than with good.

But the most valuable part of the treatment of cystttis is the use of laudanum and starch in the rectum. Let from 20 to 60 drops of tincture of opium be mixed with two ounces of starch mucilage, and thrown into the rectum with a syringe. This enema may be repeated two or three times a day. Those unacquainted with the quieting effects of this agency, in irritation of the bladder and cystitis, will be happily surprised when they carry the plan into operation. No internal medication through the stomach can equal, in curative effects, these sedatives and emollient enemas. In addition, a bag of hot sand may be placed between the thighs, near the perineum, and a hot dinner-plate may be frequently placed upon the hypogastrium. By medicating the pelvic viscera and surroundings, the stomach may be kept for food and drink. Sedative medicines injure the appetite and digestion. Run as few remedies through the stomach as possible, unless they be peptics.—*Eclectic Medical Journal.*

**An Effectual Antidote to Bad Air.**—Dr. Goulden, of London, in a communication to the *Leeds Mercury*, gives a very simple method of purifying air which is so offensive as to be totally unfit for breathing. He says it has been tried on small and on very large scales, and never failed in its results. It is used in hospitals, large school-rooms, slaughter houses, tanners' yards, the lower decks of ships, where several hundred seamen have made the atmosphere so impure during the night as to be most offensive to any one from the outside suddenly entering. It is reasonable, and based upon the laws of physical science, and so simple that one of our best practical chemists said: "I wonder that it never occurred to us before." It is just the application of a very weak solution of chloride of lead, suspended in the atmosphere on a towel in the room, and in considerably less than one minute the air is deprived of the slightest trace of anything offensive or deleterious. The room may be of any dimensions, and so long as there is any lead in the solution there cannot exist any of the poisonous gas in the atmosphere; in fact, the confined air of a hospital becomes much purer than that of any large town, as may be proved by its failing to tarnish the most delicate silver work or silver lace after months of exposure.

The mode of using it is: Take a drachm of nitrate of lead and dissolve it in a pailful of soft water, and take a drachm of common salt, which dissolve in a jug of soft water, and when the solutions are mixed it is ready for use.—*Braithwaite's Retrospect.*

**The Monobromide of Camphor as a Hypnotic.**—Frankhauser recommends the monobromide of camphor as a hypnotic in cases in which the narcotics proper are not borne, or in which they have lost their efficacy from long use. The doses required to produce sleep (he gives generally 2 to 2⅓ grains in powder) are entirely harmless; unpleasant effects—such as a feeling of fulness in the head, nausea, excitement—occur but rarely, and even then are only transitory. In most cases the drug is well borne, even though gastric catarrh, cardialgia, etc., exist. The hypnotic effect follows rapidly, as a rule; and in many cases, even when the drug has been used repeatedly, lasts for a considerable time. In other cases it passes off in a relatively short period. In some cases these small doses refused to produce sleep, but it is probable that larger doses would have been more successful.—Three and three-quarter grains, and even more, have been given without injury.—*Deutsche Medical Woch.*

**On the Use of Carbolic Acid in Surgical Diseases.**—Bryant saw severe collapse occur in two cases, as a result of the external application of carbolic acid. In one of these cases he had used a twenty per cent. solution of the acid in oil for a slight wound, and in the other ten per cent. watery solution to dress a resection of the hip. Both cases recovered after the acid was discontinued; the urine soon became clear again. Hence he discards carbolic acid as a surgical dressing. Howse, however, claims that Bryant erred in using such strong solutions of the acid. He has used it for seven years in the proportions recommended by Lister (five or ten per cent. for carbolic oil, two-and-a-half to three per cent. for the watery solution used as an irrigating fluid), and has never met with any alarming symptoms. The discoloration of the urine, which appeared in some of his cases, disappeared without treatment, and without necessitating the discontinuance of the dressing. He does not consider it necessary to stop the use of the acid unless albuminuria should set in, in addition to the discoloration of the urine.—*Guy's Hospital Report*, 1877.

**Supra-Pubic Lithotomy.**—The bladder should not be distended with an injection. "The operation in its simplest form is conducted as follows: The skin just above the pubes and over the linea alba is incised to the extent of a few inches, and an easy dissection brings on down to the region of the bladder. This is now pushed up on the end of a sound, passed through the urethra, and secured with a tenaculum. It is then incised to a proper extent, and the calculus removed with fingers or forceps. After which the wound should be covered with a light absorbent and stimulating dressing, the patient put to bed, and the subsequent treatment conducted on general principles."

The method of raising the bladder on the sound should be practiced first, if possible, on the cadaver. Theoretically, this operation affords the most direct, easy, simple and safe access to the bladder, and the author has no doubt that if performed as generally as the perineal section, the results would be far more satisfactory.—*N. Y. Med. Journal.*

**Acute Carbolic Acid Poisoning—Recovery.**—Oberst (*Cbl. f. Chir.* from *Berlin, Klin. Wochens.*, 1878, No. 12) was called to see a man who three minutes previously had swallowed five ounces of a five per cent. watery solution of carbolic acid. The patient was unconscious; a cold sweat over the face; the jaw tightly clenched. Oberst opened the œsophagus, passed the stomach-sound without difficulty, and emptied the stomach of some four ounces of its contents, which smelled strongly of carbolic acid. Several pints of water were then introduced, the stomach washed out, and the patient rapidly recovered. The urine was colored brown for one day, while a catarrh of the bladder, from which the patient had suffered, improved greatly under the influence of the carbolic acid. (Here is a hint for the treatment of vesical catarrh which deserves to be followed out.)—*Transcript.*

**Incipient Diphtheria.**—A person with headache, deep muscular and periosteal pains, sore throat, with diphtheritic patches on the tonsils, is in need of medicine, but is commonly in no danger. Such a patient may be treated as follows: ℞ Syrup of ginger f℥ij., chloral hydrate ʒj., tinct. veratrum gtt. x. M. Dose half a teaspoonful every two hours. A piece of flannel is put around the neck; the diet is restricted to slops, and the sufferer kept quiet and comfortable.—*Eclectic Med. Journal.*

**The Jacques Catheter.**—The employment of the Jacques Catheter, as recommended by Dr. Butcher and myself, is among the greatest of the minor offices of surgery. The instrument is eminently fitted for evacuating the bladder when inability to micturate exists. Many a man just passed the middle of life has said to me: "That catheter has saved my life, or has obviated the pain of a thousand deaths." The instrument will not cure cancer of the bladder, nor remove a large vesical calculus, but it can be employed by the patient himself whenever he feels a desire to micturate, so that he does not have to send for the doctor, and be severely tortured with an unyielding metallic instrument. Once I dreaded to treat those old urinary difficulties. Now it is a source of happiness to feel that I can afford substantial relief to that class of cases.—H., in *Eclectic Med. Journal.*

**Injecting Pile Tumors With Carbolic Acid**—Within a year or two a new method of treating chronic hemorrhoids has been introduced. At first the operation was kept a secret, and the manner of operating was sold for considerable sums of money. Now the manner of proceeding is known to everybody in the profession. It consists in injecting each prominent pile tumor with a dilute solution of carbolic acid; the common hypodermic syringe being the vehicle for the introduction of the fluid. Two or three tumors may be injected during one operation. The pain is not severe, nor the after suffering intolerable. The operations may be repeated once a week until the tumors have disappeared. Two or three operations will generally result in a satisfactory cure. Proper systemic treatment hastens a favorable result. The old plan of excising and ligating hemorrhoidal protrusions is attended with too much danger to be repeated, now that a safer plan has been invented.—*Ibid.*

**Treatment of Puerperal Convulsions.**—Dr. Denham, in some sensible remarks on this subject before the Dublin Obstetrical Society, says, the varied causes indicating puerperal convulsions should never be forgotten; thus a plethoric woman, with rapid pulse, flushed face, constipated bowels, should not be treated in the beginning with chloral. A good dash of blood from the patient's arm, together with free purgation, besides filling an obvious indication, prepares the way for anæsthesia, by choral or chloroform. He believes that chloral injected acted much more rapidly than when given by the mouth.—*Toledo Medical Journal.*

**Cactus and Heart's-Ease.**—Cactus and heart's-ease act on the heart and are useful in heart disease. Lungwort acts on the lungs, and is useful in lung disease. Tincture of arnica, in very small doses, will stop bleeding at the lungs. Prussiate of potash will cicatrize the lungs, if ulcerated, and relieve the cough. Balsam of tolu is healing to the lungs, and when combined with tincture of myrrh, syrup of senega and squill, will cure almost any cough that is curable.—*Ibid.*

**Myrica and Subnitrate of Bismuth.**—Myrica and subnitrate of bismuth combined will cure diarrhœa and catarrh of the head, because they act on the mucous glands, and arrest secretion, and they also act directly on the part affected.—*Eclectic Med. Journal.*

